# Resilience at the core: transforming primary health care in Latin America and the Caribbean

**DOI:** 10.1016/j.lana.2025.101274

**Published:** 2025-10-10

**Authors:** The Lancet Regional Health – Americas

Our current understanding of health as a human right is founded in the Alma-Ata Declaration from 1978, which also defined primary health care (PHC) as the key to achieving health for all. Although solidified as essential, access to PHC can be impaired by circumstantial and unpredictable events, such as a pandemic or extreme weather events like hurricanes and floods, leaving people unassisted. The ability to maintain core functions in unfavourable and disruptive circumstances, known as resilience, is also a well established concept in public health, but is usually considered separate from PHC. In this October issue, we publish the final report from the World Bank–PAHO–Lancet Regional Health Americas Commission *No time to wait: resilience as a cornerstone for primary health care across Latin America and the Caribbean*, with a central and powerful message: the development of PHC-based systems and health system resilience are synergistic, mutually reinforcing, and an urgent matter for the Latin American and Caribbean (LAC) region.

PHC, which includes early diagnosis, treatment, rehabilitation, and palliative care, is the most accessible and cost-effective level of care. A strong PHC system acts as the first line of defence, preventing minor health issues from escalating into complex and costly conditions that require higher-level interventions. Considering PHC as the foundational level of a health system is not only cost-saving but also the route for universal health access. However, this also means that any disruption to PHC (eg, a pandemic, natural disaster, or political instability) can have far-reaching consequences for entire populations and building a resilient system becomes imperative.

LAC is the world's most unequal region. Resulting from a complex composite, the roots of inequity in the LAC region go beyond the high-income disparities and intergenerational economic persistence, to include gender and ethnic-based disparities and historical exclusions inherited from colonisation. Altogether, the LAC region is positioned as an extremely vulnerable region where unavoidable disruptions can have devastating effects on millions of lives, and the most vulnerable are the first ones to be affected. In a modelling prediction, the impact from a single disruptive event causing a 25–50% reduction in primary care coverage in the LAC region could range from US$7 billion to more than $35 billion in economic losses, and tens of thousands of additional deaths, including up to 11,300 maternal deaths and 131,000 deaths from non-communicable diseases. Those are preventable deaths, provided we transform our health systems to be able to anticipate, absorb, and recover from inevitable shocks in the future.

Historically, reforms aimed at building PHC-centred systems in the LAC region had unsatisfactory outcomes, mostly due to an absence of sustained prioritisation across political cycles and insufficient financial support. As a result, most countries continue to struggle with fragmented, underfinanced, and inefficient systems that fail to provide basic care for the population. To put resilience in the core might seem obvious, especially after the COVID-19 pandemic, but this integrated vision, in which PHC is the foundation and the driver of resilience, represents a paradigm shift for health care systems in the LAC region.

To guide this transformation, the Commission sets out five high-level recommendations that include strengthening comprehensive and equitable models of care that provide services for all across the resilience cycle, integrating essential public health functions into PHC, fostering community empowerment and trust, establishing multisectoral action and policies, and ensuring sustainable and resilient PHC financing. However, an even more important recommendation is for countries to evaluate where they stand in the transformation pathway. Some might already have a PHC-based system and can focus on building resilience, others might be far behind in ensuring basic level coverage for the population. The Commission provides a comprehensive policy framework that serves as a practical tool for countries to assess their current systems, identify gaps, and prioritize interventions that will strengthen resilience across the entire cycle. The journey must start with listening to communities, understanding local vulnerabilities, and prioritising actions that are both feasible and impactful. This self-assessment should guide the implementation of a context-based plan with the overarching goal to achieve a fully resilient PHC-centered system designed by and for the people it serves. A crucial and frequently overlooked aspect in this framework is the empowerment of the community, which should be considered with a gender-based and race-based lens, to ensure that frequently excluded voices are considered and a truly inclusive system is built. This is especially urgent in the LAC region, where deep-rooted inequities magnify the impact of each crisis.

By being embedded in communities, PHC is uniquely positioned to address the social determinants of health, build trust, and respond to the diverse needs of populations. Therefore, perhaps the most innovative and important contribution of this Commission report is to be entirely produced based on the LAC context, building on local evidence collated by regionally based researchers in a massive collaboration across the countries that compose the region, taking into consideration community workers' voices, policy makers, and academic expertise to build contextualized, evidence-based guidance to advance health-care provision for all.

This Commission report is more than a technical document, it is a call to action for leadership. In an era of rising geopolitical tension, growing science scepticism, and advancing climate crisis, the time to act is running out.

